# [^18^F]SiTATE-PET/CT for detection of pheochromocytomas and paragangliomas: comparison of biochemical secretion, genotype and imaging metrics

**DOI:** 10.1007/s00259-025-07341-9

**Published:** 2025-05-30

**Authors:** Meike Onkes, Paul Dahlmann, Alena Gesenhues, Sean Ira G. Gacula, Nabeel Mansour, Júnia R. O. L. Schweizer, Isabel Stüfchen, Christian Lottspeich, Katharina Wang, Matthias K. Auer, Matthias Brendel, Alessa Fischer, Till Braunschweig, Franz-Josef Gildehaus, Simon Lindner, Christine Schmid-Tannwald, Thomas Pfluger, Christoph J. Auernhammer, Svenja Nölting, Rudolf A. Werner, Martin Reincke, Martin Bidlingmaier, Matthias Kroiss, Friederike Völter

**Affiliations:** 1 Department of Nuclear Medicine, LMU University Hospital, LMU Munich, 81377 Munich, Germany; 2 Department of Medical Imaging and Therapeutic Radiology, National Kidney and Transplant Institute, Quezon City, Manila Philippines; 3 Department of Radiology, LMU University Hospital, LMU Munich, 81377 Munich, Germany; 4 Department of Medicine IV, Endocrinology, Diabetes and Metabolism, LMU University Hospital, LMU Munich, 80336 Munich, Germany; 5 German Center for Neurodegenerative Diseases (DZNE), 81377 Munich, Germany; 6 German Cancer Consortium (DKTK), Partner Site Munich, a partnership between German Cancer Research Center (DKFZ) and LMU Munich, Munich, Germany; 7 Bavarian Cancer Research Center (BZKF), Partner Site Munich, Munich, Germany; 8 Munich Cluster for Systems Neurology (SyNergy), Munich, Germany; 9 Department of Endocrinology, Diabetology and Clinical Nutrition, University Hospital Zurich (USZ) and University of Zurich (UZH), 8091 Zurich, Switzerland; 10 Institute of Pathology, LMU University Hospital, LMU Munich, 80337 Munich, Germany

**Keywords:** SSTR, [^18^F]SiTATE, PET/CT, PPGL, Paraganglioma, Pheochromocytoma, Molecular imaging, Catecholamines, LCMS, ELISA

## Abstract

**Background:**

Somatostatin-receptor (SSTR)—targeting PET/CT is widely used for diagnosis and disease monitoring of pheochromocytoma / paraganglioma (PPGL). The aim of this study was to assess the potential of the novel SSTR-targeting tracer [^18^F]SiTATE in diagnosing PPGL by comparing imaging parameters to tumor marker levels and secretory activity in a small cohort of patients diagnosed with this rare tumor type.

**Methods:**

This retrospective study included 34 patients with histologically confirmed PPGL who underwent [^18^F]SiTATE-PET/CT at LMU University Hospital Munich between 10/2020 and 02/2024 as well as hormonal laboratory analysis within up to 100 days. Imaging parameters — standardized uptake values (SUVmax, SUVmean), metabolic tumor volume (MTV), and total lesion uptake (TLU) — were analyzed. Uptake was normalized to liver background (SUVmaxr, SUVmeanr). Radioligand uptake of biochemical subtypes and genotypes was compared with Mann-Whitney-U test. Correlation was tested using Spearman´s rank correlation test.

**Results:**

The patient-based detection rate of [^18^F]SiTATE-PET was 96.6%. A moderate correlation was found between MTV and TLU with chromogranin A (r = 0.570-0.608, p < 0.005) and with biochemical secretion (r = 0.466-0.576, p < 0.05). Hereditary PPGL with Cluster 1 genotype showed stronger [^18^F]SiTATE uptake compared to sporadic PPGL (SUVmeanr: p = 0.032; SUVmaxr: p = 0.051). A subgroup comparison with [⁶⁸Ga]Ga-DOTATOC-PET/CT revealed no significant difference in uptake metrics or tumor-to-background ratios.

**Conclusion:**

This is the first clinical evaluation of [^18^F]SiTATE-PET/CT in patients with PPGL. MTV and TLU measured with [^18^F]SiTATE-PET/CT correlated well with the tumor marker chromogranin A in serum and with (nor)metanephrines in urine and plasma. Within the limits imposed by the small cohort, our results suggest that TLU and MTV in [^18^F]SiTATE-PET/CT could be used as SSTR imaging biomarker for monitoring of disease progression and secretory activity in patients with PPGL.

**Supplementary Information:**

The online version contains supplementary material available at 10.1007/s00259-025-07341-9.

## Introduction

Paragangliomas and pheochromocytomas (PPGL) are rare neuroendocrine tumors that originate from chromaffin cells in the adrenal medulla or neural crest-derived progenitor cells located outside the adrenal gland in the prevertebral and paravertebral sympathetic ganglia of the chest, abdomen, and pelvis or the parasympathetic ganglia (head and neck paragangliomas) [1]. Some PPGL are biochemically active. These tumors are characterized by their production of catecholamines (epinephrine, norepinephrine, dopamine). Primary diagnostics include the laboratory quantification of their metabolites (metanephrine, normetanephrine, 3-methoxytyramine in plasma or metanephrine and normetanephrine in urine). In addition, tumor marker chromogranin A is quantified [2–5].

Biochemical secretory patterns, which are also associated with distinct genotypes, allow for the characterization of PPGL into three different biochemical phenotypes. Tumors with a noradrenergic phenotype predominantly produce norepinephrine (normetanephrine), whereas those with an adrenergic phenotype predominantly produce epinephrine (metanephrine) together with or without norepinephrine. A third, rare biochemical phenotype produces predominantly dopamine (assessed by 3-methoxytyramine) [6–11]. In the current literature, the noradrenergic and dopaminergic subtype are frequently grouped together as a single entity [12].

Previously thought to have a hereditary basis in only about 10% of cases, recent studies have shown that a significantly larger proportion, approximately 25–40%, are indeed linked to genetic syndromes in the caucasian population [13–19]. Based on the underlying genetic alterations, PPGL can be classified into three molecular clusters [12]: Cluster 1A includes pathogenic variants (PV) in genes associated with the tricarboxylic acid cycle, such as the succinate dehydrogenase gene family (SDHx). Cluster 1B comprises PV in genes related to von Hippel-Lindau disease (VHL) or endothelial PAS domain 1 (EPAS1) [12]. Cluster 2 encompasses PV in genes linked to tyrosine kinase signaling pathways, including the RET proto-oncogene, neurofibromin 1 (NF1) and MYC-associated factor X (MAX) [12]. Each molecular cluster is associated with a distinct biochemical phenotype: Cluster 1A/B PPGLs frequently present with noradrenergic phenotypes while Cluster 2 PPGLs are associated with adrenergic phenotypes [12, 14]. Additionally, Cluster 1-related PPGLs are associated with a higher metastastic risk than Cluster 2 PPGLs [12].

For tumor localization, tumor burden, assessment of metastases and of potential radioligand therapy, functional imaging with positron emission tomography/computed tomography (PET/CT) is performed pre- or postoperatively following conventional imaging via computed tomography (CT) or magnetic resonance imaging (MRI) [20, 21]. Among these techniques, somatostatin receptor PET/CT enables visualization of the overexpression of somatostatin receptors specific to neuroendocrine tumors [22, 23].

In recent years, the novel radioligand [^18^F]SiTATE has been introduced for somatostatin receptor PET/CT [22, 24, 25]. [^18^F]SiTATE, a SiFAlin tagged [Tyr3]-octreotate (TATE) firstly enables the use of the radionuclide fluorine-18 ([^18^F]). The adoption of [^18^F] allows for more cost-effective, and more reliable production of radioligands for somatostatin receptor PET/CT compared to the previously used radionuclide gallium-68 ([^68^Ga]) [26, 27]. Additionally, [^18^F] provides a higher image resolution than [^68^Ga] due to favorable physical properties of the radionuclide.

[^18^F]SiTATE-PET/CT has been validated in several clinical studies for various neuroendocrine tumor types, particularly gastroenteropancreatic neuroendocrine tumors (GEPNET) [28–31]. However, [^18^F]SiTATE has not yet been evaluated for the rarer neuroendocrine tumors such as PPGL. Thus, this retrospective study aims to compare imaging parameters on [^18^F]SiTATE-PET/CT, specifically tracer uptake and metabolic tumor burden, with the biochemical secretion and the genotype of PPGL, to determine its potential as a diagnostic tool for these rare neuroendocrine tumors.

## Materials and methods

### Patient inclusion

All patients with PPGL who underwent PET/CT imaging with [^18^F]SiTATE at LMU University Hospital Munich between October 2020 and February 2024 and with a laboratory analysis at LMU University Hospital Munich within up to 100 days from the PET scan were included in this retrospective analysis. Patients with a PPGL manifestation limited to the head-neck region, were excluded from the study, as they often are biochemically silent [32, 33]. All patients were referred for imaging by their respective endocrinologists and provided written informed consent to undergo [^18^F]SiTATE-PET/CT in compliance with the regulations of the German Pharmaceuticals Act. The study was conducted in accordance with the principles of the Declaration of Helsinki and its subsequent amendments [34]. Approval from the local ethics committee was obtained (approval number 379–10). All patients provided written informed consent to participate in the study. The metastatic status was defined imaging-based at the time of the PET/CT scan. Multiple primaries were not considered as metastatic disease.

### PET/CT imaging and postprocessing

SiTATE was obtained from ABX, Advanced Biomedical Compounds (Radeberg, Germany), and [^18^F]SiTATE was synthesized as previously described [26, 29]. All quality control data met the release criteria. All [^18^F]SiTATE-PET/CT scans were acquired at the Department of Nuclear Medicine, LMU Munich, using a GE Discovery™ PET/CT 690.

After intravenous injection of [^18^F]SiTATE, PET data were acquired 90 min post-injection with 2.5 min per scan position including the head, the neck, the thoracic region, the abdomen and the pelvis. All patients were premedicated with furosemide (20 mg/2 mL injection solution, ratiopharm GmbH, Ulm, Germany) for radiation protection, provided there were no medical contraindications [35]. Contrast-enhanced CT scans were acquired with 1.5 mL of iopromide (Ultravist 300, Bayer Healthcare, Leverkusen, Germany) per kilogram of body weight, unless there were no medical contraindications. PET/CT scans were performed with 40–200 mAs, 120 kV, collimation 2 × 5 mm, pitch of 0.976. A transaxial 512 × 512 matrix and the Enhancement Hanning filter was used to reconstruct the PET images, which were reconstructed with the VPFX (= OSEM with TOF) algorithm.

### Image analysis

Image analysis was performed using a dedicated software package (Hermia Hybrid Viewer, Affinity 1.1.4, Hermes Medical Solutions, Stockholm, Sweden). The radioligand uptake of the tumor lesions was assessed with maximum and mean standardized uptake value (SUVmax and SUVmean). The background activity was assessed using a 3 cm spherical volume of interest (VOI) placed in spleen, mediastinal blood pool of the left ventricle and in a non-tumorous area of the liver. For normalization of the tumor uptake, ratios of tumor SUV to SUVmean of the liver were calculated for SUVmax (SUVmaxr) and SUVmean (SUVmeanr). Metabolic tumor volume (MTV) was assessed semimanually using a SUV-threshold of 5.0 within a manually predefined VOI. Organs with physiological radioligand uptake were excluded manually from the segmentation. Total lesion uptake (TLU) was calculated by multiplication of the metabolic tumor volume and SUVmean of the latter one (MTV x SUVmeanr). The radioligand avidity of tumor lesions was assessed by two PET/CT readers, one board-certified specialist in nuclear medicine and one specialist in radiology. The PET/CT readers had access to the patients'previous radiological scans, including previous CT and MRI scans, if needed for the assessment. The number of tumor lesions on the PET/CT was assessed for all tumor lesions and separately for the primary/local recurrence, bone, lymph node, liver, pulmonary and thyroid metastases as well as for peritoneal carcinomatosis. Bone, lymph node and liver metastases were counted as 20, if a patient had more than 20 bone, lymph node or liver metastases, respectively. Tumor lesions were considered PET-positive if the radioligand uptake was visually increased compared to the background uptake. Three PET-negative bone metastases were not included in this analysis because they were considered nonviable after external beam radiotherapy.

### Laboratory analysis

Metanephrine and normetanephrine in plasma were analyzed by Enzyme-Linked Immunosorbent Assay (2-MET plasma ELISA fast track, LDN, Nordhorn, Germany) in the endocrine diagnostics laboratory of the Department of Medicine IV, LMU Munich [36]. Normetanephrine, metanephrine and 3-methoxytyramine (3MTyr) in plasma were additionally analyzed by Liquid Chromatography-Mass Spectrometry (LCMS). Urinary metanephrines were determined in 24-h urine samples by high-performance liquid chromatography at the laboratory of LMU University Hospital Munich. Serum chromogranin A (CgA) was determined by ELISA (Cisbio) according to the manufacturer’s instructions at the laboratory of LMU University Hospital Munich. Prior to blood sampling, patients were placed in the supine position for a duration of 20 min to ensure adequate and standardized preanalytical conditions for the procedure. The samples were immediately stored on ice until measurement if performed within 6 h. Otherwise, the samples were stored at −20 °C.

### Assessment of the genetic cluster

The genetic cluster was assessed with a Next Generation Sequencing panel in clinical routine in 27 cases and for research purposes in 1 case. No testing for somatic pathogenic variants was included in this analysis.

### Definition of biochemical secretion type

The biochemical phenotype of the PPGL was defined using the plasma concentration of metanephrine and normetanephrine. If the plasma concentration of metanephrine was above 62 pg/mL (0.31 nmol/L) and there was a tumor-derived increase of metanephrine of more than 5% of the combined increases of normetanephrine and metanephrine, patients were classified with the adrenergic phenotype [37]. All other tumors are defined as noradrenergic/dopaminergic (hereafter referred to as noradrenergic), including those with elevations of plasma 3-methoxytyramine [12].

### Statistical analysis

Data analysis was performed using Microsoft Excel (Excel 2019, Microsoft, Redmond, WA) and SPSS software (IBM SPSS Statistics 29, Armonk, NY). Descriptive statistics are displayed as median with interquartile range (Q1, Q3) or mean ± standard deviation (SD) depending on whether they showed normal distribution according to the Shapiro–Wilk test. For binary variables, PET/CT parameters and laboratory values were compared using Mann-Whitney-U test. For the comparison of radioligand uptake in patients with different genotype and biochemical phenotype, only patients with detectable tumor volume on CT were included. When metanephrine or chromogranin A concentrations were below the detection limit, random values between zero and the corresponding detection limit were generated with a random number generator (Excel 2019, Microsoft, Redmond, WA). Spearman’s correlation analysis was used for numerical variables to compare MTV and TLU with biochemical secretion. A log transformation was applied to normalize the skewed data of plasma and urinary biochemical measurements for spearman correlation and graphic visualization. A significance level of p < 0.05 was applied in all analyses. For the comparison of the correlation of different measurements, Fisher’s z transformation was used. The graphics were created using Graphpad Prism (GraphPad Prism 8.4.3).

## Results

### Study population

After exclusion of 5 patients with PPGL limited to the head-neck region, a total of 34 patients were enrolled, comprising 20 patients with pheochromocytoma, 13 patients with paraganglioma and 1 patient with both. 17 patients were male, 17 female, the median age was 55 years (range 5–83). Detailed characteristics of the study population are presented in Table 1.

**Table 1:** Biochemical and clinical characteristics of the study population

Total number of included patients	34
Age at PET/CT scan [years]	55 ± 17
Sex: male/female	17 (50.0%)/17 (50.0%)
**diagnosis**	*n*
pheochromocytoma	20 (58.8%)
paraganglioma	13 (38.2%)
pheochromocytoma + paraganglioma	1 (2.9%)
**genetic background**	*n*
not available	6 (17.6%)
sporadic*	15 (44.1%)
Cluster 1A	11 (32.4%)
Cluster 1B	2 (5.9%)
**positive germline PV**^**1**^	*n*
overall	13
SDHA	1 (2.9%)
SDHB	8 (23.5%)
SDHC	1 (2.9%)
SDHD	1 (2.9%)
VHL	2 (5.9%)
**Ki-67 of the primary**	*n*
patients with available Ki-67	17
≤ 2%	5 (14.7%)
2–20%	3 (8.8%)
> 20%	9 (26.5%)
n. a	17 (50%)
median Ki-67 (Q1; Q3)	10% (2%; 20%)
**biochemical subtype**	*n*
adrenergic	8 (23.5%)
noradrenergic/dopaminergic	26 (76.5%)
**pretreatment**	*n*
no prior therapy	10 (29.4%)
surgery	23 (67.6%)
chemotherapy	3 (8.8%)
[^177^Lu]Lu-DOTA-TATE therapy	8 (23.5%)
[^131^I]MIBG therapy^2^	6 (17.6%)
external beam radiation	6 (17.6%)
brachytherapy liver	1 (2.9%)
anti-resorptive therapy	5 (14.7%)
**tumor localization**	
**localized disease**	8 (23.5%)
**metastatic disease**	21 (61.8%)
primary/recurrence of primary	9 (26.5%)
bone	15 (44.1%)
liver	4 (11.8%)
lymph nodes	14 (41.2%)
lung	2 (5.9%)
peritoneal	1 (2.9%)
thyroid	1 (2.9%)
no detectable tumor**	5 (14.7%)
**Dose-length-product** [mGy*cm]	915 ± 496
**Computed tomography dose index** [mGy]	9.8 ± 3.9

*The genetic testing excluded germline PVs in these patients, somatic PVs were not tested

**No detectable tumor was identified on the computed tomography scan, and the laboratory analysis revealed normal levels of tumor markers and catecholamines

^1^PV = pathogenic variant

^2^[^131^I]MIBG = [^131^I]meta-iodobenzylguanidine

Most patients had undergone prior treatments before PET/CT scan (mean interim time in month ± SD), including surgery (n = 23; 90.9 ± 88.9), chemotherapy (cyclophosphamide, vincristine and dacarbazine, n = 3; 26.4 ± 26.0) [38], radioligand therapy with [^177^Lu]Lu-DOTA-TATE (n = 8; 16.6 ± 13.3), [^131^I]meta-iodobenzylguanidine (MIBG) therapy (n = 6; 62.0 ± 43.3), external beam radiation therapy (n = 6; 43.1 ± 28.5), brachytherapy of liver metastases (n = 1; 23.3), and anti-resorptive therapy with denosumab (n = 5; 16.7 ± 12.0). Ten patients had no prior treatment.

15 patients (44.1%) had negative germline testing and were therefore considered sporadic cases. Eleven patients (32.4%) had SDHA (n = 1), SDHB (n = 8), SDHC (n = 1) and SDHD (n = 1) germline PV and were therefore considered as patients with PPGL of the Cluster 1A subtype. Two patients (5.9%) had a VHL PV and were therefore considered as cases with PPGL of the Cluster 1B genotype. For six patients (17.6%), no data about genetic testing was available. Somatic PVs were not tested.

21 patients had metastatic disease at the time of the PET/CT scan. The sites of the metastatic disease were bone (n = 15), liver (n = 4), lymph nodes (n = 14), lung (n = 2), peritoneum (n = 1) and thyroid (n = 1). Five patients had no local recurrence or metastasis after primary surgery.

The median time between the PET/CT scan and the collection of the laboratory hormone secretion by LCMS-test was 1 day (Q1 = 0 day; Q3 = 11 days), by ELISA-test was also 1 day (Q1 = 0 day; Q3 = 6 days) and urinary test was 8 days (Q1 = 0 day; Q3 = 9 days). Laboratory results of the study population are presented in Table 2. Based on the biochemical secretion (see above), the study population was divided into adrenergic subtype (n = 8) and noradrenergic subtype (n = 26).

**Table 2:** Biochemical parameters of all included patients. Values are presented as median (Q1; Q3). The number of patients with available results from each respective parameter is displayed in the second column

	**n**		**upper reference limit (URL)**
**Serum chromogranin A [ng/ml]**	29	221 (126; 1380.5)	≤ 101
**Plasma metanephrines (LCMS)**	25		
metanephrine [ng/l]		34 (24; 58)	< 90
normetanephrine [ng/l]		328 (136.5; 616)	< 200
3-methoxytyramine [ng/l]		12 (4.5; 35)	< 28
**Plasma metanephrines (ELISA)**	28		
metanephrine [pg/ml]		41.5 (20; 90.25)	< 100
normetanephrine [pg/ml]		200.5 (92.25; 568.75)	< 216
**Urinary parameters**	20		
metanephrine [mg/l]		0.72 (0.26; 1.52)	
metanephrine [mg/24 h]		0.92 (0.43; 2.16)	
norepinephrine [µg/l]		36.8 (27.6; 95.6)	
norepinephrine [mg/24 h]		82.9 (43.9; 189)	≤ 100
epinephrine [µg/l]		3.1 (0.5; 5)	
epinephrine [mg/24 h]		6.1 (1.3; 13.4)	≤ 20
dopamine [µg/l]		118 (88.9; 137)	
dopamine [mg/24 h]		253 (150; 293)	≤ 500
normetanephrine [µg/l]		396.5 (208; 1110.75)	
normetanephrine [mg/24 h]		718 (360; 1872)	≤ 311

### Radioligand uptake of PPGL lesions on [^18^F]SiTATE PET/CT

**Fig. 1:**
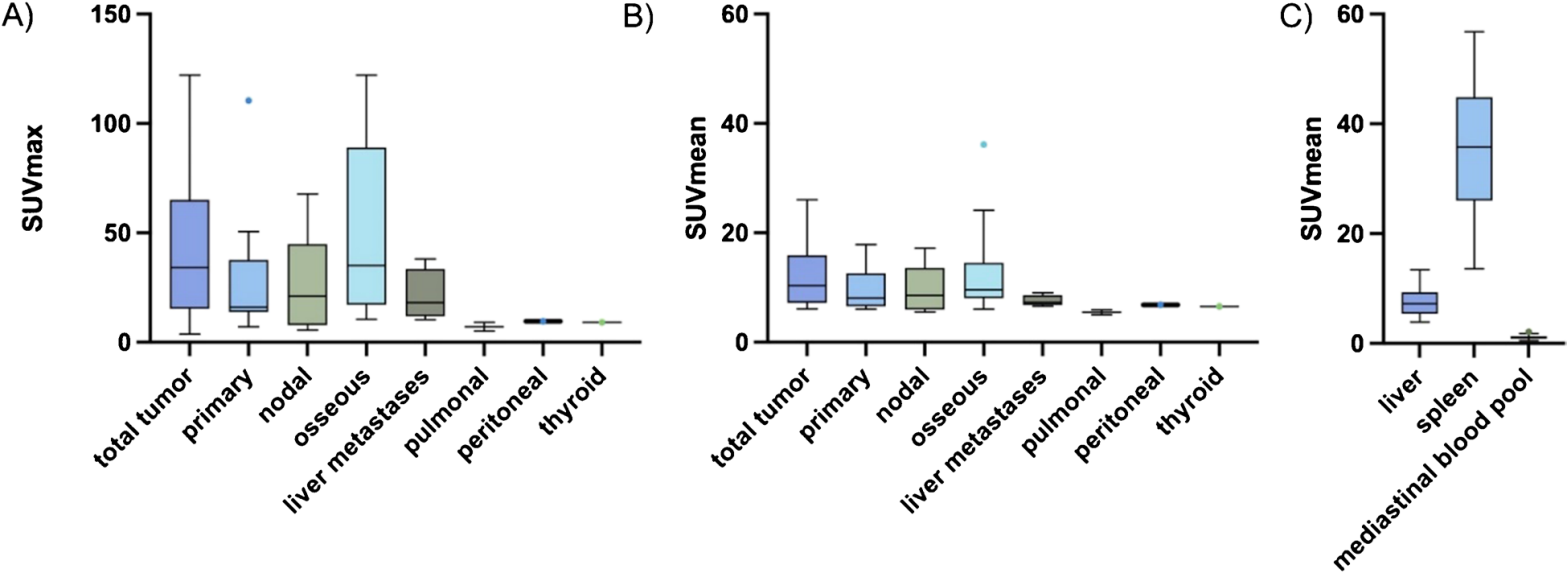
Boxplots of mean [^18^F]SiTATE uptake intensity (SUVmax and SUVmean) in PPGL lesions and physiological reference regions, stratified by metastasis type. (**A**) Mean SUVmax and (**B**) mean SUVmean of residual tumor lesions in 29 patients. (**C**) Physiological uptake in healthy liver, spleen, and mediastinal blood pool in all 34 included patients

**Fig. 2:**
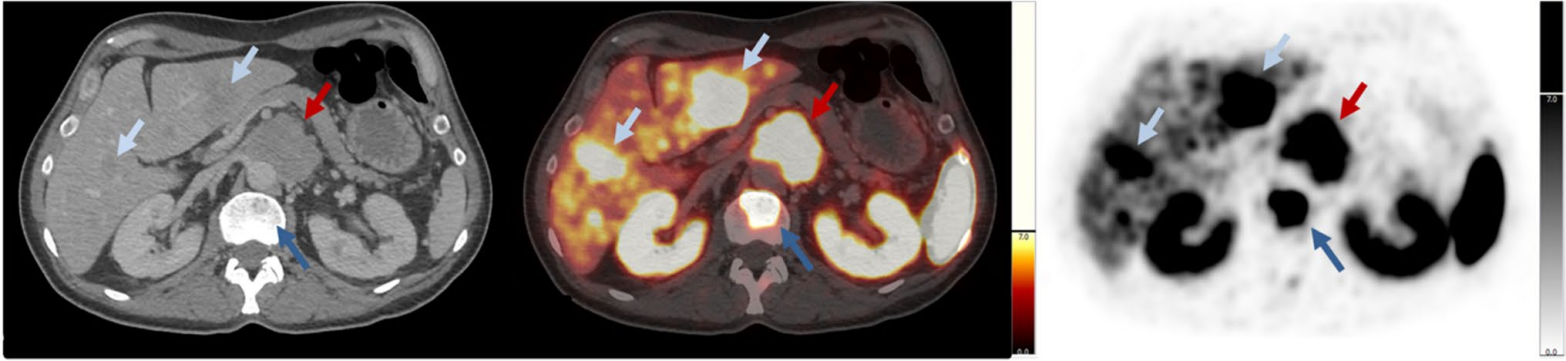
[^18^F]SiTATE-PET/CT showing distinct tracer uptake in a 51-year-old male patient with untreated sporadic paraaortic paraganglioma and metastatic disease. Displayed are axial CT (soft tissue window), axial PET/CT (soft tissue window), and axial PET images. Red arrow: primary paraaortic lesion; light blue arrows: liver metastases; dark blue arrow: osseous metastasis

The mean injected dose of the radioligand [^18^F]SiTATE was 219 ± 49 MBq. Contrast-enhanced CT scans were acquired in 30 out of 34 patients. The remaining four patients underwent diagnostic CT scans without contrast enhancement due to a contrast allergy (n = 1) or low-dose CT scans without contrast enhancement as they had recently undergone a diagnostic CT scan with contrast (n = 3). 28 of 29 patients with visible tumor on CT presented with radioligand avid PPGL lesions on [^18^F]SiTATE-PET/CT, achieving a patient-based true positive rate of 96.6%. In total, 347 tumor lesions were evaluated with 22 primaries/local recurrences (2 patients with multiple primaries), 79 lymph node metastases, > 168 bone metastases, > 66 hepatic metastases, 20 pulmonary metastases, one peritoneal and one thyroid metastasis. Two lesions of two different patients were PET-negative, one primary and one pulmonary metastasis with a CT diameter of 2 mm. The patient without discernible tracer uptake of the primary (SUVmax = 3.6; SUVmean = 2.5; SUVmaxr = 0.46; SUVmeanr = 0.32; MTV = 0 ml; TLU = 0) was female and had a non-metastasized sporadic right-sided pheochromocytoma with 2.8 × 2.6 × 2.6 cm with an adrenergic phenotype. An immunohistochemical staining of the histopathological specimen with SSTR 2A showed completely absent staining (Volante score 0) (Supplementary Fig. 1). Evaluating the intensity of radioligand uptake of tumorous lesions, the strongest radioligand uptake of [^18^F]SiTATE was observed in bone metastases, followed by the radioligand uptake of the primary/local recurrence, lymph node metastases and liver metastases. The lowest uptake intensity was observed in pulmonary metastases. A peritoneal and a thyroid metastasis showed a moderate radioligand uptake, respectively. The quantitative radioligand uptake of the primary and different types of metastases is presented in Table 3 and Fig. 1. Figure 2 shows a [^18^F]SiTATE-PET/CT scan of a patient with a paraganglioma and metastases in the liver, bones, lymph nodes and lungs.

**Table 3:** Uptake intensity of PET-positive PPGL lesions on [^18^F]SiTATE-PET/CT stratified across type of metastasis. SUVmaxr = SUVmax of the tumor/SUVmean of the liver; SUVmeanr = SUVmean of the tumor/SUVmean of the liver; MTV = metabolic tumor volume [ml]; TLU = total lesion uptake (MTV x SUVmeanr). Values are reported as mean ± SD or as individual numeric values (peritoneal, thyroid)

region	primary/recurrence	bone metastases	lymph node metastases	liver metastases	pulmonal metastases	peritoneal metastasis	thyroid metastasis
number of patients	**n = 16**	**n = 15**	**n = 14**	**n = 4**	**n = 2**	**n = 1**	**n = 1**
uptake intensity SUVmax [mean ± SD]	27.80 ± 25.56	68.95 ± 104.94	26.85 ± 22.36	21.15 ± 11.92	7.05 ± 2.90	9.6	9.1
uptake intensity SUVmaxr [mean ± SD]	4.57 ± 4.58	10.48 ± 14.23	4.31 ± 4.03	3.64 ± 1.22	1.52 ± 0.84	0.98	1.59
uptake intensity SUVmean [mean ± SD]	9.72 ± 3.92	13.03 ± 8.21	9.72 ± 4.19	7.53 ± 1.03	5.45 ± 0.64	6.8	6.5
uptake intensity SUVmeanr [mean ± SD]	1.55 ± 0.85	2.14 ± 1.07	1.59 ± 0.80	1.40 ± 0.26	1.15 ± 0.32	0.69	1.14

#### Background radioligand uptake of healthy organs

There was a low physiological uptake of the mediastinal blood pool (mean SUVmean 1.12 ± 0.35), a moderate radioligand uptake of the non-tumorous liver (mean SUVmean 7.42 ± 2.55), and a strong radioligand uptake of the healthy spleen (mean SUVmean 35.81 ± 11.22) (see also Fig. 1).

#### Tracer uptake of hereditary and sporadic PPGL

**Fig. 3:**
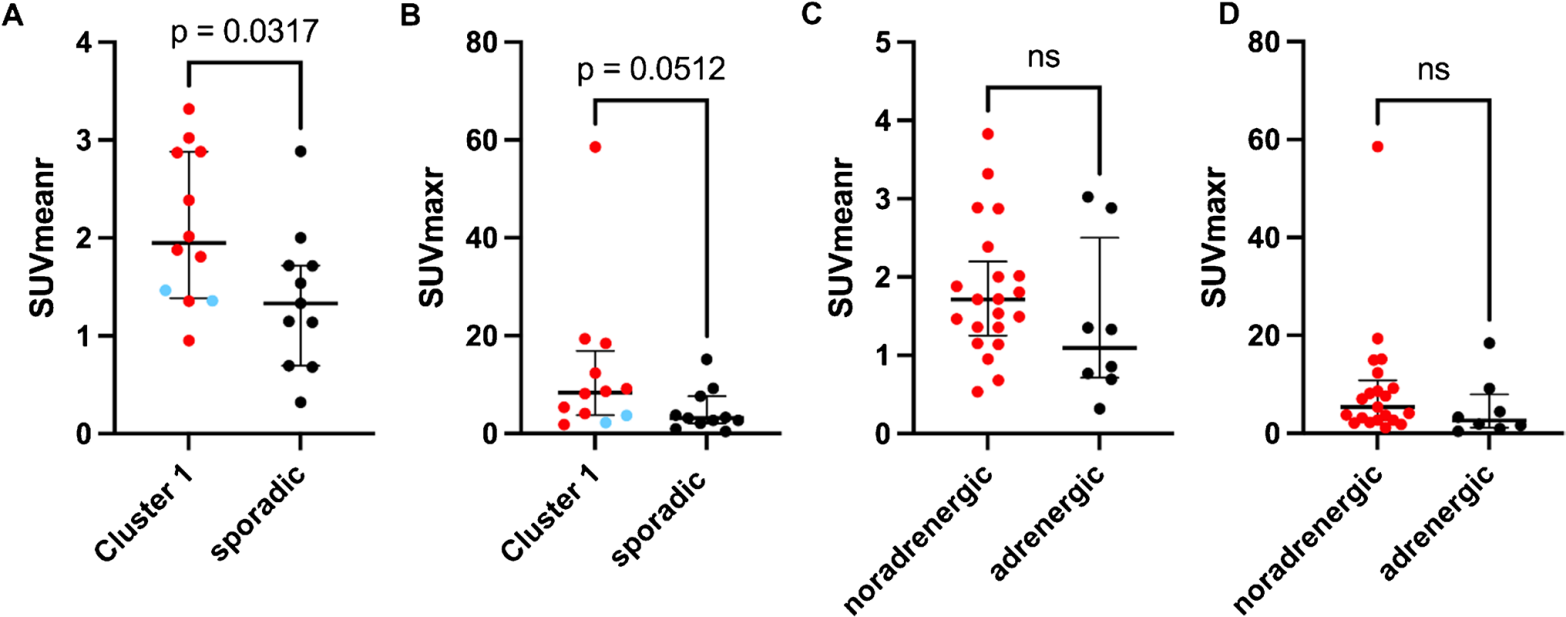
Uptake intensity of PPGL with different genotypes (**A**, **B**) and biochemical phenotypes (**C**, **D**). A and B: Mann-Whitney-U test showed a significantly increased SUVmeanr and non-significantly elevated SUVmaxr of Cluster 1 PPGL compared to sporadic PPGL. Within the column of Cluster 1, the data points for Cluster 1A PPGL (all with SDHx pathogenic variants) are marked with red dots, and the data for Cluster 1B PPGL are marked with blue dots. C and D: Mann-Whitney-U test showed no significant difference between uptake intensity of noradrenergic and adrenergic PPGL. Dopaminergic PPGL were grouped with noradrenergic PPGL

MTV and TLU of patients with hereditary and sporadic PPGL are displayed in Table 4 and Fig. 3. Mann–Whitney-U test showed an increased uptake intensity in Cluster 1 PPGL (median SUVmaxr: 8.39 vs. 3.19, p = 0.051; median SUVmeanr: 1.95 vs. 1.33, p = 0.032), Cluster 1A PPGL (median SUVmaxr: 8.90 vs. 3.19, p = 0.020; median SUVmeanr: 2.20 vs. 1.33, p = 0.016) and in patients with SDHB PV (median SUVmaxr: 10.49 vs. 3.19, p = 0.026; median SUVmeanr: 2.20 vs. 1.33, p = 0.041) compared to sporadic PPGL. Additionally, compared to the MTV and TLU of sporadic PPGL, MTV and TLU were non-significantly increased in Cluster 1 (median TLU: 73.29 vs. 13.05, p = 0.525; median MTV: 37.40 vs. 9.90, p = 0.740), Cluster 1A (median TLU: 133.91 vs. 13.05, p = 0.511; median MTV: 62.85 vs. 9.90, p = 0.756), and in patients with SDHB PV (median TLU: 133.90 vs. 13.05, p = 0.657; median MTV: 62.90 vs. 9.90, p = 0.717). Radioligand uptake of Cluster 1B was not compared to the other groups due to the small sample size. A subgroup analysis was conducted for the patients with metastatic disease (Supplementary Table 1). Similarly, there was a significant difference of SUVmaxr (p = 0.009) and SUVmeanr (p = 0.012) between metastatic sporadic PPGL (n = 10) and metastatic Cluster 1 PPGL (n = 8) and no significant difference comparing TLU (p = 0.573) and MTV (p = 0.897). There was a non-significant positive correlation between Ki-67-index and SUVmaxr (r = 0.44, p = 0.079) and SUVmeanr (r = 0.41, p = 0.099). No correlation was found between Ki-67 and MTV or TLU (p = 0.330 or p = 0.320).

**Table 4:** [^18^F]SiTATE uptake across different genotypes and biochemical phenotypes. All 29 patients with detectable tumor on CT were included. SUVmaxr = SUVmax of the tumor/SUVmean of the liver; SUVmeanr = SUVmean of the tumor/SUVmean of the liver; MTV = metabolic tumor volume [ml]; TLU = total lesion uptake (MTV x SUVmeanr). Values are reported as median (Q1; Q3) or as individual numeric values (Cluster 1B). Custer 1A contained 8 patients with SDHB pathogenic variants (PV), one patient with SDHA PV and one patient with SDHD PV. Cluster 1B contained 2 patients with VHL PV. Genetic testing was not available for 6 of the 29 patients

	overall	noradrenergic subtype	adrenergic subtype	sporadic	Cluster 1 (1A + 1B)	Cluster 1A	SDHB		Cluster 1B
number of patients	**n = 29**	**n = 21**	**n = 8**	**n = 11**	**n = 12**	**n = 10**	**n = 8**	**n = 2**	
SUVmaxr	4.11 (2.16; 9.21)	5.34 (2.69; 10.80)	2.62 (1.15; 7.97)	3.19 (2.12; 7.62)	8.39 (3.80; 16.89)	8.90 (5.03; 18.64)	10.49 (6.04; 19.12)	3.70 and 2.21	
SUVmeanr	1.49 (1.05; 2.20)	1.71 (1.25; 2.20)	1.09 (0.71; 2.50)	1.33 (0.69; 1.72)	1.95 (1.39; 2.88)	2.20 (1.69; 2.92)	2.20 (1.49; 2.88)	1.36 and 1.47	
TLU	46.06 (10.16; 579.54)	49.60 (12.20; 579.54)	14.53 (2.45; 568.55)	13.05 (4.09; 924.65)	73.29 (21.28; 486.02)	133.91 (37.06; 568.72)	133.90 (47.0; 486.0)	24.57 and 20.19	
MTV [ml]	26.30 (6.45; 217.00)	34.00 (8.75; 250.00)	10.35 (1.90; 210.00)	9.90 (3.60; 569.00)	37.40 (14.78; 164.75)	62.85 (21.13; 198.00)	62.90 (28.2; 164.8)	18.00 and 13.70

#### Tracer uptake of different biochemical subtypes

SUVmeanr, SUVmaxr, TLU and MTV were increased in patients with the noradrenergic phenotype compared to patients with the adrenergic phenotype. However, statistical significance was not reached (median SUVmaxr: 5.34 vs. 2.62, p = 0.153; median SUVmeanr: 1.71 vs. 1.09, p = 0.139; median TLU: 49.60 vs. 14.53, p = 0.279; median MTV: 34.00 vs. 10.35, p = 0.279). See also Fig. 3 PET parameters of patients with different biochemical subtypes are displayed in detail in Table 4.

#### Comparison of [^18^F]SiTATE-PET/CT and previous [^68^Ga]Ga-DOTATOC-PET/CT in patients with PPGL

**Fig. 4:**
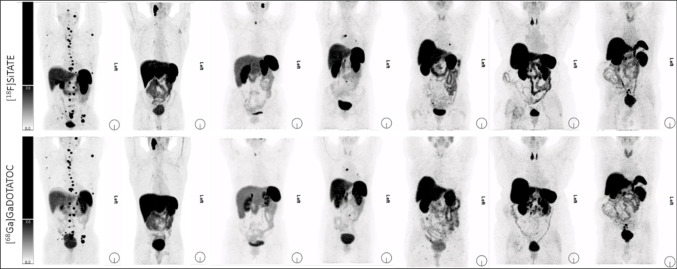
Comparison of [^18^F]SiTATE-PET/CT and previous [^68^Ga]Ga-DOTATOC-PET/CT in 7 patients with near-stable disease and no treatment between the two PET scans. Maximum intensity projections of [^18^F]SiTATE-PET (upper row) and [^68^Ga]Ga-DOTATOC-PET (lower row)

Overall, in 15 of the included patients, a previous [^68^Ga]Ga-DOTATOC-PET/CT was available, with a median interval of 11 months (Q1: 12.3; Q3: 8.7). All tumor lesions, that showed an increased radioligand uptake on the previous [^68^Ga]Ga-DOTATOC-PET/CT also showed increased radioligand uptake on the follow-up [^18^F]SiTATE-PET/CT. 6 patients presented with progressive disease at the follow-up [^18^F]SiTATE-PET/CT. A subgroup of 7 patients presented with near-stable disease status and no tumor specific treatment in the meantime, 2 patients showed no disease on the [^68^Ga]Ga-DOTATOC-PET/CT and the follow-up [^18^F]SiTATE-PET/CT. The median interval of the two PET/CT scans was 12 months (Q1: 29.1; Q3: 10.8) in this subgroup. A mean activity of 234 ± 43 MBq was applied for the [^68^Ga]Ga-DOTATOC-PET/CT imaging and a mean activity of 215 ± 21 MBq for the [^18^F]SiTATE-PET/CT. On [^18^F]SiTATE-PET/CT, a non-significantly increased radioligand uptake of both, PPGL lesions and healthy tissue was noted (Table 5). There was no significant difference between MTV and TLU measured on [^18^F]SiTATE-PET/CT compared to [^68^Ga]Ga-DOTATOC-PET/CT. Maximum intensity projections of [^18^F]SiTATE-PET/CT and [^68^Ga]Ga-DOTATOC-PET/CT of all 7 patients with near-stable disease are presented in Fig. 4; Further axial PET/CT slices are presented in Supplementary Fig. 2.

**Table 5:** Comparison of uptake characteristics of the total tumor volume and background uptake in patients with PPGL undergoing watch and wait without specific treatment and with detectable tumor on CT (n = 7). SUVmaxr = SUVmax of the tumor/SUVmean of the liver; SUVmeanr = SUVmean of the tumor/SUVmean of the liver; MTV = metabolic tumor volume [ml]; TLU = total lesion uptake (MTV x SUVmeanr)

Total tumor volume	SUVmax [mean ± SD]	SUVmaxr [mean ± SD]	SUVmean [mean ± SD]	SUVmeanr [mean ± SD]	MTV [median;1^st^quartile, 3^rd^quartile]	TLU [median;1^st^quartile, 3^rd^quartile]	liver [SUVmean]	spleen [SUVmean]	blood pool [SUVmean]
[^18^F]SiTATE	36.19 ± 29.97	4.20 ± 3.88	10.66 ± 3.97	1.24 ± 0.52	5.60 (4.45; 21.95)	6.70 (4.59; 28.07)	9.23 ± 2.31	34.44 ± 10.40	1.19 ± 0.40
[^68^Ga]Ga-DOTATOC	33.94 ± 28.23	4.02 ± 3.96	9.67 ± 3.26	1.15 ± 0.47	5.10 (3.50; 33.10)	6.89 (3.56; 38.63)	8.46 ± 2.36	25.74 ± 6.57	0.98 ± 0.32
level of significance	*p* = *0.710*	*p* = *1.00*	*p* = *0.535*	*p* = *1.00*	*p* = *0.710*	*p* = *0.805*	*p* = *0.489*	*p* = *0.065*	*p* = *0.297*

### Correlation of metabolic tumor volume and total lesion uptake with the tumor marker chromogranin A

**Fig. 5:**
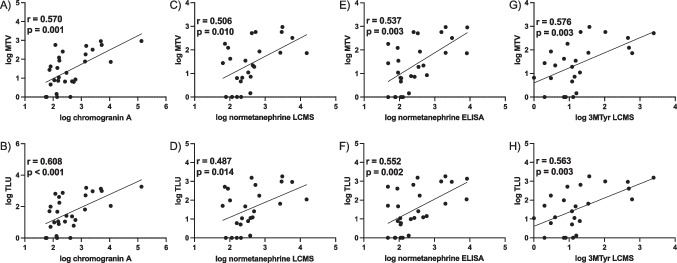
Correlation between biochemical secretion and metabolic tumor volume (MTV) (upper row) and total lesion uptake (TLU) (lower row) on [^18^F]SiTATE-PET/CT. There was a moderate positive correlation between chromogranin A in plasma and MTV (A) and TLU (B). There was also a moderate correlation of MTV and TLU with normetanephrine in plasma measured with LCMS (C—D) and ELISA (E—F) and with 3-methoxytyramine (3MTyr) measured with LCMS (G—H)

Spearman’s rank correlation analysis was performed to determine a correlation between MTV and TLU on [^18^F]SiTATE-PET/CT with hormone secretion and the tumor marker chromogranin A. There was a significant correlation of the tumor marker chromogranin A with MTV (r = 0.570, p = 0.001) and TLU (r = 0.608, p < 0.001). Details are presented in Table 6 and Fig. 5. In the presented cohort, six patients were taking a proton pump inhibitor regularly. The (partial) correlation between MTV, TLU and chromogranin A remained significant when adding proton pump inhibitor pantoprazole intake as a confounding covariable (MTV: r = 0.434, p = 0.021; TLU: r = 0.435, p = 0.021).

**Table 6:** Spearman’s rank correlation analysis of MTV and TLU on [^18^F]SiTATE-PET/CT with biochemical secretion of PPGL. All values were log-transformed before analysis. Significant results (p < 0.05) were highlighted

Spearman correlation		normetanephrine		3MTyr* LCMS	24 h-urinary norepinephrine	24 h-urinary dopamine	chromogranin A
		LCMS	ELISA				
MTV [ml]	r *p*	**0.506** **0.010**	**0.537** **0.003**	**0.576** **0.003**	**0.511** **0.025**	0.409 0.082	**0.570** **0.001**
TLU	r *p*	**0.487** **0.014**	**0.552** **0.002**	**0.563** **0.003**	**0.569** **0.011**	**0.466** **0.044**	**0.608** ** < 0.001**

*3MTyr = 3-methoxytyramine; LCMS = Liquid Chromatography–Mass Spectrometry; ELISA = Enzyme–Linked Immunosorbent Assay

### Correlation of the metabolic tumor volume and the total lesion uptake with biochemical secretion

**Fig. 6:**
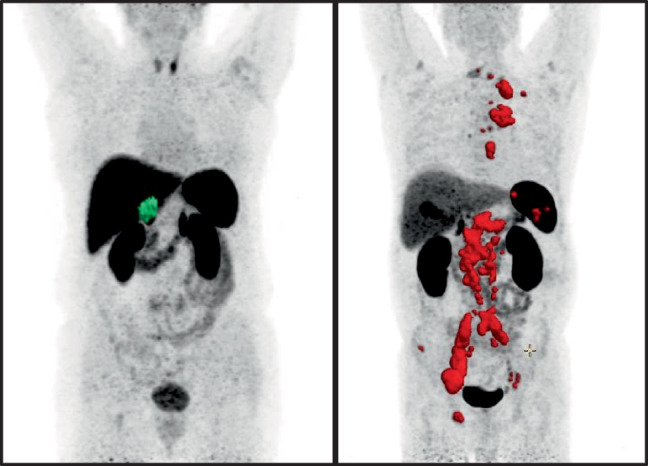
[^18^F]SiTATE-PET/CT in two PPGL patients with limited (left) and metastatic disease (right)

The MTV correlated significantly with plasma normetanephrine (r = 0.506, p = 0.010) and plasma 3MTyr (r = 0.576, p = 0.003) in the LCMS measurement, with plasma normetanephrine (r = 0.537, p = 0.003) in the ELISA measurement and with 24 h-urinary norepinephrine (r = 0.511, p = 0.025), respectively. Comparison of the strength of the correlation using a z-transformation showed that the correlation of normetanephrine levels was not significantly different between LCMS and ELISA measurements (p = 0.442 and p = 0.380). TLU correlated significantly with normetanephrine (r = 0.487, p = 0.014) and 3MTyr (r = 0.563, p = 0.003) in LCMS test. Another significant correlation was between TLU and normetanephrine in ELISA test (r = 0.552, p = 0.002). Statistically significant correlations were also observed between TLU and the value of 24 h-urinary norepinephrine (r = 0.569, p = 0.011) and dopamine (r = 0.466, p = 0.044). There was no significant correlation between MTV, TLU and plasma metanephrine, plasma epinephrine, 24 h-urinary metanephrine, 24 h-urinary epinephrine and 24 h-urinary normetanephrine. Table 6 displays the correlation analysis for all hormones that showed significant correlations with MTV and TLU. The complete analysis with all hormones analyzed and with non-significant correlations is displayed in Supplementary Table 2. The correlation between plasma catecholamines and serum chromogranin A with MTV and TLU is shown in Fig. 5**.** Selective measurement of the correlation for patients with either the noradrenergic or adrenergic phenotype did not enhance the strength of the correlation between biochemical secretion and MTV (data not shown). Patient examples of [^18^F]SiTATE-PET/CT in two PPGL patients with different extents of tumor disease and metastatic status are displayed in Fig. 6**.**

Left: 75-year-old female patient with pheochromocytoma of the right adrenal gland (semi-automated metabolic tumor volume (MTV) segmentation highlighted in green); Image parameters: MTV = 18 ml, TLU = 24.6; chromogranin A = 295 ng/ml, normetanephrine in ELISA = 343 pg/ml. Right: 57-year-old male patient with pheochromocytoma of the left adrenal gland with lymph node, bone and peritoneal metastases (semi-automated MTV segmentation highlighted in red); Image parameters: MTV = 321 ml, TLU = 924.65; chromogranin A = 2,498 ng/ml, normetanephrine in ELISA = 3,117 pg/ml.

## Discussion

This is the first study investigating the novel SSTR-PET tracer [^18^F]SiTATE in patients with PPGL, which offers logistical and economic advantages over [^68^Ga]-labeled radioligands. Our findings demonstrate a high rate of PET-positive PPGL and a strong tumor-to-background ratio using [^18^F]SiTATE. MTV and TLU correlated moderately but significantly with the tumor marker chromogranin A and with the biochemical secretion, supporting the utility of PET/CT as a diagnostic tool for PPGL. Cluster 1 PPGL and SDHB-related PPGL showed a significantly increased radioligand uptake compared to sporadic PPGL, highlighting a potential stratification approach for imaging.

PPGL are classified based on their primary location, biochemical phenotype and genetic cluster. These factors, along with metastatic status and planned treatment – such as radioligand therapy – inform the choice of the diagnostic radionuclide for PET/CT imaging. In patients with metastatic disease, SSTR-PET/CT is preferred over [^18^F]DOPA-PET/CT (if the genetic background is not known), as metastatic disease is associated with the noradrenergic phenotype and SSTR-PET also allows for a pretherapeutic assessment of tumor uptake before radioligand therapy, which is critical for therapy success. These considerations are reflected in our patient cohort, where a significant proportion presented with metastases. Additionally, two thirds of the cohort showed a noradrenergic phenotype, which is—as stated above—also linked to metastatic disease.

The patient-based detection rate of [^18^F]SiTATE in our study cohort (96.6%) is comparable to reported detection rates of [^68^Ga]-labeled SSTR-targeting radioligands in the literature: A metaanalysis from 2022 including 13 studies with 215 patients with PPGL of all genotypes showed a pooled detection rate of 93% (95% CI, 91%–95%) of [^68^Ga]DOTA–conjugated SSTR-targeting PET with no significant difference in the detection rate across different radioligands [39]. In non-metastasized sporadic pheochromocytoma, [^18^F]DOPA-PET/CT is the radioligand of choice [40]. In our study cohort, 20 patients with pheochromocytoma were included, of whom 19 were true positive on [^18^F]SiTATE-PET/CT while one patient with a non-metastatic pheochromocytoma showed false negative results. Concordantly, immunohistochemical (IHC) staining showed no SSTR 2A expression in this patient. A previous study of the ENS@T network investigating IHC expression of SSTR 2A in 202 patients with PPGL including 101 SSTR-IHC negative tumors showed, that non-metastatic PPGL were more frequently SSTR-IHC negative compared to metastatic PPGL [41].

In our cohort, patients with SDHx and SDHB PV showed stronger radioligand uptake on [^18^F]SiTATE-PET/CT than sporadic PPGL. An increased SSTR 2A-IHC staining of patients with SDHx-related PPGL (n = 18) compared to sporadic PPGL (n = 41) was previously shown [41]. Previous imaging studies with [^68^Ga]DOTATATE showed an increased sensitivity of SSTR-PET in patients with SDHx PV [42–45]: Kong et al. showed a 100% per patient sensitivity in 20 patients with SDHx-related PPGL [42]. Janssen et al. showed a 98.6% lesion-based sensitivity in 17 patients with SDHB-related PPGL [43].

A subgroup comparison of [^18^F]SiTATE-PET/CT and previous [^68^Ga]Ga-DOTATOC-PET/CT scans showed no significant difference in radioligand uptake of PPGL lesions, healthy organs and no significant difference of the tumor-to-background ratio. This finding indicates that [^18^F]SiTATE-PET/CT can be equally effective as [^68^Ga]Ga-DOTATOC in the imaging of PPGL patients. In accordance with our findings, a previous study on [^18^F]SiTATE-PET/CT in meningiomas demonstrated a non-significantly increased radioligand uptake of tumor and background on [^18^F]SiTATE-PET/CT compared to [^68^Ga]Ga-DOTATOC-PET/CT as well [30].

A moderate correlation was observed between MTV on [^18^F]SiTATE-PET/CT and serum chromogranin A levels, with chromogranin A being commonly used as a tumor marker for neuroendocrine tumors. However, it is important to note, that chromogranin A levels are subject to multifactorial regulation. Its levels can be influenced by various factors, including the intake of certain medications, such as proton pump inhibitors, and a wide range of comorbidities, such as renal impairment, infections, COPD, gastritis, rheumatic arthritis, irritative bowel disease, hypertension or heart failure [46, 47]. Moreover, chromogranin A levels are more frequently elevated in well-differentiated than in poorly differentiated neuroendocrine tumors [47]. These factors underscore the complexity of interpreting chromogranin A levels in clinical practice and their correlation with imaging biomarkers.

When comparing biochemical secretion to MTV and TLU on [^18^F]SiTATE-PET/CT, the strongest correlations were observed with plasma normetanephrine and 3-methoxytyramine, as well as norepinephrine and dopamine in 24 h urine. To evaluate whether the strong correlation with normetanephrines was influenced by the predominance of PPGL with the noradrenergic phenotype in our study cohort, we separately assessed correlations of adrenergic and noradrenergic phenotypes. However, excluding adrenergic PPGL did not enhance the correlation between normetanephrine levels and MTV or TLU. This suggests that the observed correlation with normetanephrines is not solely due to the higher proportion of noradrenergic PPGL in our cohort.

Previous studies using [^18^F]DOPA-PET/CT have similarly reported significant correlations between biochemical secretion and PET parameters [48–50]. For instance, in a cohort of 39 patients with pheochromocytomas, the total lesion uptake on [^18^F]DOPA-PET/CT demonstrated robust correlations with 24 h urinary excretion of normetanephrine (r = 0.64, p < 0.0001) and metanephrine (r = 0.49, p = 0.002), as well as with plasma free levels of normetanephrine (r = 0.55, p = 0.006) [49]. Furthermore, an analysis of 42 patients with PPGL found that MTV correlated strongly with normetanephrines in plasma and urine (r = 0.82–0.84) and moderately with urinary metanephrines (r = 0.57) [50]. Additionally, an earlier study involving 56 patients with PPGL observed a strong positive correlation of MTV and urinary metanephrines (r = 0.80, p < 0.001) [48]. Taken together, our findings, combined with those from the existing literature, suggest that tumor volume and total lesion uptake are strongly correlated with the absolute amount of catecholamine secretion, irrespective of the biochemical phenotype.

Compared to [^68^Ga]-labelled SSTR-receptor targeting PET/CT tracers, the conjugation with [^18^F] allows for a higher spatial resolution and more logistic flexibility due to the longer half-time. The production of [^18^F] is independent of a Gallium-Germanium generator and allows for larger-badge productions. The feasibility of the new tracer [^18^F]SiTATE for SSTR imaging of neuroendocrine tumors was already shown for patients with medullary thyroid carcinoma and for patients with GEPNET [28, 29, 31]. The results of our study expand the applicability of [^18^F]SiTATE to the PET/CT imaging of PPGL.

The small sample size of our study limits the statistical power of our findings. However, given the rarity of PPGL, the sample size of our study remains meaningful and is comparable to those in other studies on functional imaging in PPGL. The retrospective design of the study led to incomplete laboratory data, as not all values were available for every patient and the majority of patients had received previous therapies before PET-CT scan that could potentially influence hormone secretion. Furthermore, the small number of Cluster 1B PPGL and the absence of Cluster 2 and 3 PPGL restricts the generalizability of our findings to these subgroups.

Future prospective studies with larger, more diverse cohorts, including patients from all genetic clusters, are necessary to fully validate the utility of [^18^F]SiTATE-PET in PPGL diagnosis and staging. Another major limitation of this study is the high proportion of patients with aggressive tumors with a Ki-67 index of > 20% and with SDHB-related PPGL that does not correspond to an average patient collective of patients with PPGL, thereby limiting the generalizability of our findings.

Conclusion: This is the first study to evaluate PET/CT imaging with the novel radioligand [^18^F]SiTATE in patients with PPGL. Our findings suggest that [^18^F]SiTATE is well-suited for the diagnosis and staging of paragangliomas and pheochromocytomas with a high percentage of PET-positive tumors and a moderate correlation of tumor volume and total lesion uptake with biochemical secretion.

## Supplementary Information

Below is the link to the electronic supplementary material.

Supplementary file1 (PDF 2.50 MB)

## Data Availability

The datasets generated during and/or analysed during the current study are available from the corresponding author on reasonable request.
