# Supplementary material for: [18F]SiTATE-PET/CT for detection of pheochromocytomas and paragangliomas: comparison of biochemical secretion, genotype and imaging metrics
Source: Eur J Nucl Med Mol Imaging. 2025 May 30;52(13):5175–88. doi: 10.1007/s00259-025-07341-9 (PMC12589282; doi:10.1007/s00259-025-07341-9)
Supplement: Supplementary file 1 — Supplementary file1 (PDF 2.50 MB) [file 259_2025_7341_MOESM1_ESM.pdf]

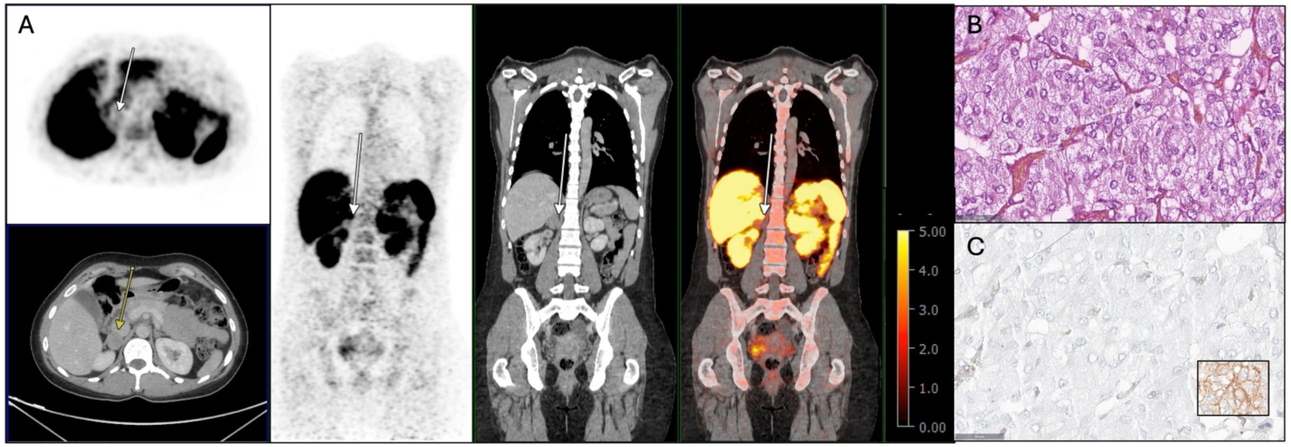

Supplementary Fig. 1 Example of a [^{18}F]SiTATE-negative pheochromocytoma

A: [^{18}F]SiTATE-PET/CT (in row: axial plane (PET, CT), coronal plane (MIP, CT, PET/CT)) of a 42 year-old female patient with pheochromocytoma of the right adrenal gland without discernible tracer uptake; Image parameters: SUVmax = 3.6; SUVmean = 2.5; SUVmaxr = 0.46; SUVmeanr = 0.32; MTV = 0 ml; TLU = 0; chromogranin A = 137 ng/ml, normetanephrine in ELISA = 125 pg/ml. B and C: Histology and Immunohistochemistry of the pheochromocytoma (400x magnification), B: hematoxylin and eosin stain showing trabecular tumor growth without significant nuclear pleomorphism or visible mitotic activity. C: immunohistochemistry for SSTR 2A at 400x magnification reveals no staining, indicating an absence of somatostatin receptor 2A expression in tumor cells. The inset in C highlights positive physiological staining in the adrenal gland. (SSTR 2A: somatostatin-receptor 2 A).

Supplementary Table 1 [^{18}F]SiTATE uptake across different genotypes in metastatic status. SUVmaxr = SUVmax of the tumor/SUVmean of the liver; SUVmeanr = SUVmean of the tumor/SUVmean of the liver; MTV = metabolic tumor volume [ml]; TLU = total lesion uptake (MTV x SUVmeanr). Values are reported as median (Q1; Q3).

	**sporadic**	**Cluster 1**
	**n = 10**	**n = 8**
**SUVmaxr**	3.26 (2.68; 6.66)	10.75 (7.81; 18.64)
**SUVmeanr**	1.44 (1.14; 1.72)	2.20 (1.86; 2.92)
**TLU**	61.43 (7.24; 855.07)	133.91 (48.72;488.57)
**MTV [ml]**	40.50 (6.93; 507.00)	62.85 (32.08; 155.25)

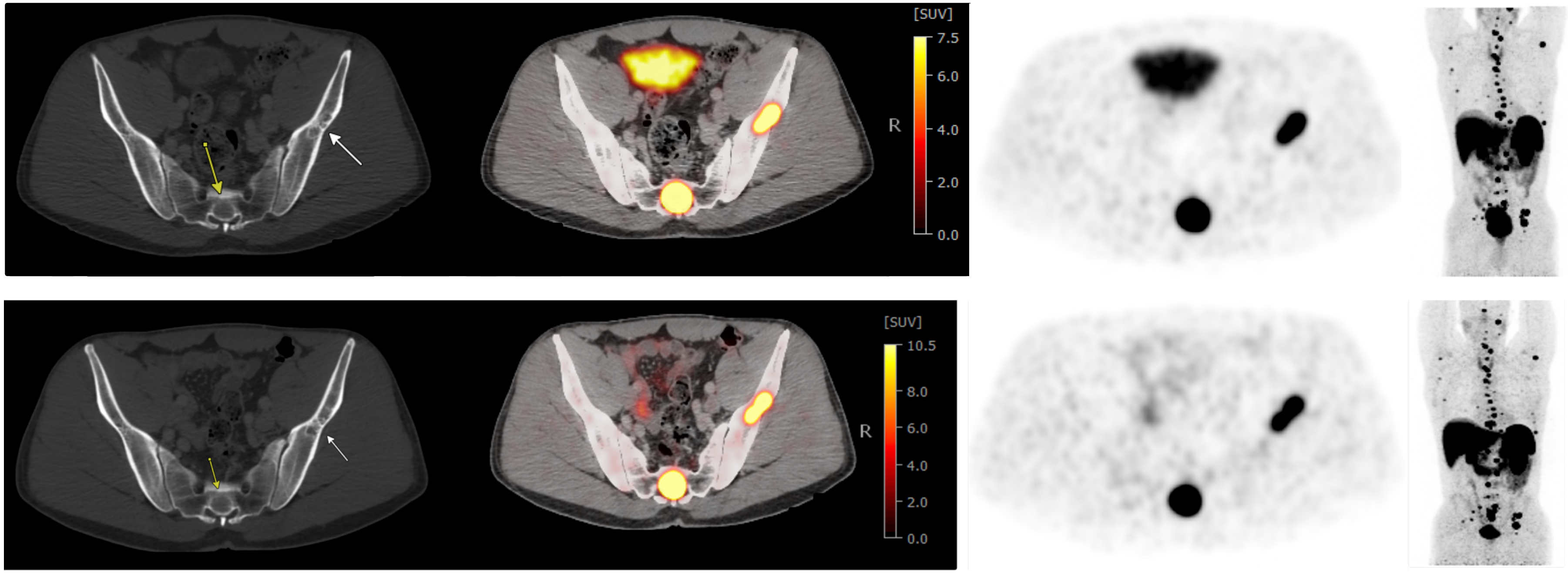

Supplementary Fig. 2 Case example of [^{18}F]SiTATE- and [^{68}Ga]Ga-DOTATOC-positive bone metastases after adrenalectomy of a right pheochromocytoma.

Upper row: [^{68}Ga]Ga-DOTATOC-PET/CT (in row: CT axial in bone window, PET/CT axial in soft tissue window, PET axial and MIP) of a 41 year-old male patient with bone metastases of a pheochromocytoma of the right adrenal gland after adrenalectomy with discernible tracer uptake; Image parameters [^{68}Ga]Ga-DOTATOC (273 MBq): SUVmax = 79.5; SUVmean = 13.1; SUVmaxr = 12.42; SUVmeanr = 2.05; MTV = 81 ml; TLU = 165.6.

Lower row: [^{18}F]SiTATE-PET/CT (in row: CT axial in bone window, PET/CT axial in soft tissue window, PET axial and MIP) of a 41 year-old male patient with bone metastases of a pheochromocytoma of the right adrenal gland after adrenalectomy with discernible tracer uptake after a periode of 11 months with watch and wait treatment; Image parameters [^{18}F]SiTATE (178 MBq): SUVmax = 88.9; SUVmean = 14.5; SUVmaxr = 12.35; SUVmeanr = 2.01; MTV = 84.9 ml; TLU = 170.83.

Supplementary Table 2 Spearman's rank correlation analysis of MTV and TLU on [^{18}F]SiTATE-PET/CT with biochemical secretion of the PPGL.
All values were log-transformed before analysis. Significant results ($p < 0.05$) were highlighted.

Spearman correlation		Plasma metanephrine	Plasma metanephrine	Plasma normetanephrine	Plasma normetanephrine	Plasma 3MTyr*	metanephrine	24h-urinary metanephrine	norepinephrine	24h-urinary norepinephrine	epinephrine	24h-urinary epinephrine	dopamine	24h-urinary dopamine	normetanephrine	24h-urinary normetanephrine	chromogranin A
		Plasma (LCMS)	Plasma (ELISA)	Plasma (LCMS)	Plasma (ELISA)	Plasma (LCMS)	Urine	Urine	Urine	Urine	Urine	Urine	Urine	Urine	Urine	Urine	Serum
**MTV [ml]**	r	0.168	0.087	**0.506**	**0.506**	**0.576**	0.049	0.168	0.372	**0.511**	0.215	0.233	0.085	0.409	0.136	0.226	**0.570**
	p	0.421	0.660	**0.010**	**0.010**	**0.003**	0.836	0.492	0.117	**0.025**	0.376	0.337	0.729	0.082	0.567	0.352	**0.001**
**TLU**	r	0.160	0.129	**0.487**	**0.487**	**0.563**	0.049	0.155	0.398	**0.569**	0.219	0.269	0.106	**0.466**	0.114	0.232	**0.608**
	p	0.444	0.512	**0.014**	**0.014**	**0.003**	0.838	0.527	0.091	**0.011**	0.367	0.266	0.666	**0.044**	0.631	0.339	**< 0.001**

*3MTyr = 3-methoxytyramine; LCMS = Liquid Chromatography – Mass Spectrometry; ELISA = Enzyme –Linked Immunosorbent Assay
